# Model Predictive Torque Control for Velocity Tracking of a Four-Wheeled Climbing Robot

**DOI:** 10.3390/s20247059

**Published:** 2020-12-10

**Authors:** Higor Barbosa Santos, Marco Antonio Simoes Teixeira, Nicolas Dalmedico, Andre Schneider de Oliveira, Flavio Neves-Jr, Julio Endress Ramos, Lucia Valeria Ramos de Arruda

**Affiliations:** 1 Graduate School of Electrical Engineering and Computer Science (CPGEI), Universidade Tecnológica Federal do Paraná (UTFPR), Curitiba 80230-901, Brazil; marcoteixeira@alunos.utfpr.edu.br (M.A.S.T.); ndalmedico@alunos.utfpr.edu.br (N.D.); andreoliveira@utfpr.edu.br (A.S.d.O.); neves@utfpr.edu.br (F.N.-J.); lvrarruda@utfpr.edu.br (L.V.R.d.A.); 2 CENPES/Petrobras, Rio de Janeiro 21941-915, Brazil; julio.ramos@petrobras.com.br

**Keywords:** model predictive control, dynamic model, climbing robot, velocity tracking, gravity, adhesion force

## Abstract

Climbing robots are characterized by a secure surface coupling that is designed to prevent falling. The robot coupling ability is assured by an adhesion method leading to nonlinear dynamic models with time-varying parameters that affect the robot’s mobility. Additionally, the wheel friction and the force of gravity force are also relevant issues that can compromise the climbing ability if they are not well modeled. This work presents a model-based torque controller for velocity tracking in a four-wheeled climbing robot specially designed to inspect storage tanks. The model-based controller (MPC) compensates for the effects of nonlinearities due to the forces of gravity, friction, and adhesion through the dynamic and kinematic modeling of the climbing robot. Dynamic modeling is based on the Lagrange-Euler approach, which allows a better understanding of how forces and torques affect the robot’s movement. Besides, an analysis of the interaction force between the robot and the contact surface is proposed, since this force affects the motion of the climbing robot according to spatial orientation. Finally, simulations are carried out to examine the robot’s dynamics during the climbing movement, and the MPC is validated through the redrobot simulator V-REP and practical experiments. The presented results highlight the compensation of the nonlinear effects due to the robot’s climbing motion by the proposed MPC controller.

## 1. Introduction

Advances in technology have increased mobile robots’ versatility and expanded their application in several areas, such as maintenance and service. Service robots are commonly designed to perform tasks in structured environments into which they must overcome different obstacles and travel through specific surfaces. Climbing robots are a particular class of service robots designed to carry out different tasks in non-horizontal surfaces, such as inspection of metal structures [1] or assisting people with physical disabilities [2].

In the literature, a significant part of motion control strategies for mobile robotics are based on kinematic models [3,4,5] that simplify or even cut out all dynamic influences. However, kinematic control can present poor performance to trajectory tracking in the presence of unknown (neglected) disturbances or unmodelled dynamics [6]. In fact, several dynamic factors can affect the robot’s motion. It is crucial to consider all these characteristics in the motion controller design, especially to climbing robots due to their varying interaction with the surface.

The adhesion force pushes the climbing robot against the surface and emphasizes the friction effect, affecting the robot’s maneuverability. The gravitational force is also a very relevant concern that cannot be neglected because it intensifies or decreases the adhesion effects due to body orientation. These aspects are commonly overlooked in 2D ground navigation since they are time-invariant. However, adhesion effects are time-varying especially, during climbing trajectories (3D navigation) through surfaces with different inclination angles.

This work develops a model-based torque controller for tracking the speed of a climbing robot. The main goal of this climbing robot is to convey a NDT (Nondestructive testing) equipment such as an ultrasound phased array during an inspection task of industrial vessels. The success of the inspection task is strongly dependent on maintaining a steady speed profile during inspection. For this, the proposed torque controller takes into account the robot’s 3D position to compensate for the effects of dynamic forces during a climbing path. In the proposed model-based torque control approach, a kinematic analysis of wheel climbing robots is firstly carried out, since an inverse kinematics model will be introduced into the dynamic model supporting the control strategy. Then, a study of the dynamics discusses the robot’s behavior to derive a reliable dynamic model for the proposed model-based controller (MPC). This study is crucial to control strategy success since it is necessary to model the effects of gravity, friction force and adhesion on the movement of the climbing robot. Dynamic modeling is based on the Lagrange-Euler method that allows a better understanding of how forces and torques affect the robot’s movement. Also, a new concept of interaction strength in dynamic modeling is discussed. The frictional force is influenced by gravity and adhesion forces according to the robot’s spatial orientation. That is, an interaction force between the contact surface and the climbing robot affects the movement according to the robot’s 3D position. Therefore, the understanding of such interaction force is an essential requirement for the dynamic modeling of the climbing robot’s motion.

## 2. Related Work

Several works discuss the dynamic modeling of mobile robots. Meanwhile only a few articles encompasses the effects of gravity, adhesion, and friction forces into the dynamic analysis of climbing robots. Dynamic modeling of a four-wheel differential robot for trajectory tracking is presented in Caracciolo et al. [7]. The proposed controller must avoid robot lateral slippage when crossing an outdoor terrain. The proposed dynamic model considers only the inertia matrix, the kinematics constraints, and the frictional force imposed on the robot. Since it is a land robot, the modeling does not consider the effects of gravity and adhesion force. Mohammadpour et al. [8] designed an adaptive controller for posture stabilization of a differential robot. The Newton-Euler approach is applied to robot dynamic modeling, but only a simple friction model is considered. Morales et al. [2] showed a climber robot to increase the mobility of people with disability in environments with architectural obstacles, such as ramps and stairs. Robot kinematic and dynamic modeling is carried out, allowing the implementation of a PD controller with compensation of nonlinear disturbances due to gravity. All these approaches are an extension of the Newton-Euler classic analysis of dynamic effects on mobile robots. The resulting models are elementary, and they do not represent the relevant characteristics of climbing robots’ dynamics.

The development of a reliable dynamic model for a robot allows the use of model-based predictive control (MPC - Model Predictive Control) techniques to compensate nonlinearities and to minimize the robot trajectory tracking error [9]. For ground robots, for example, Kuhne et al. [10] presented both a nonlinear MPC (non-convex optimization) and linear MPC (quadratic programming) for a trajectory tracking problem. When comparing the two strategies, it is observed that the linear MPC has a promising performance with a lower computational effort. However, when using a linearized model, the linear approach is only valid around points near the reference trajectory. Besides, predictive control can also be used to solve stabilization point problems. Wang et al. [11] proposed an MPC algorithm for the stabilization of points and trajectory tracking of an omnidirectional robot. Therefore, the main advantage of predictive control is the applicability in multivariate systems with multiple constraints [9].

Since the works related to climbing robots do not consider the dynamic effects of gravity, friction, and the adhesion system during robot climbing, this work contributes to bridge this gap by developing a new MPC approach to torque control of a four-wheeled climbing robot. The developed model-based torque controller considers the dynamic effects of the cited forces in the dynamic model used for speed tracking error minimization. Although the control approach is designed for a differential climbing robot, the proposed approach can be applied in other climbing robot architecture.

## 3. Overview of Climbing Robots

Climbing robots are specially designed for climbing surfaces and navigating in non-parallel ground planes. Thus, they suffer different influences of gravitational force according to their relative position. The robot dynamically interacts with the surface, aiming to nullify the gravitational effects and move in any direction without losing contact with the surface. The dynamic interaction between robot and surface is specified as adhesion, which consists of an attractive force between the parts in contact, i.e., robot locomotion system and surface.

This kind of mobile robot is characterized by its adhesion and locomotion mechanisms [12], which are specified in accord to its task, e.g., inspection of buildings [13], cleaning of glass-curtain walls [14] and fire fighting in residential towers [15]. Several combinations of locomotion system and adhesion methods can be implemented. The most common types of locomotion systems to climbing tasks are arms/legs [16], wheels [17], wired/rail [18] and tracks [19]. Main adhesion mechanisms are magnetic [20,21,22] and electrostatic [23,24], but there exist other adhesion methods, such as pneumatic [25], mechanical [26] and chemical [27]. Moreover, the non-ground tasks impose crucial requirements about weight, maneuverability, and adhesion to the robot [12,28], as discussed below.

- Weight: the robot’s mass directly affects its ability to climb since gravity disturbs the robot’s motion. Thus, the robot must be light. Furthermore, a lightweight robot allows a better efficiency of energy, which improves its navigational autonomy;
- Maneuverability: the robot’s ability to easily change its direction and position is directly affected by its locomotion and adhesion mechanisms;
- Adhesion: the adhesion mechanisms must guarantee the adherence in different surfaces, ensuring that the climbing robot does not fall during its navigation.

**Figure 1:**
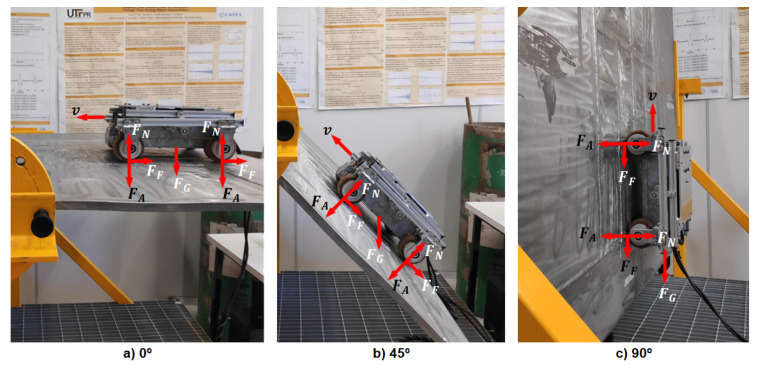
Force analysis between a climbing robot and the contact plane (wall) with different inclination angles: (**a**) 0${}^{\circ }$ wall (**b**) 45${}^{\circ }$ wall (**c**) 90${}^{\circ }$ wall. The forces present in the figure are: $F_{G}$ is the gravity force, $F_{F}$ is the frictional force, $F_{N}$ is the normal force, and $F_{A}$ is the adhesion force.

The adhesion force should always keep the robot in contact with the surface and prevent its slippage or fall. This effect is traditionally achieved by the use of magnetic or electrostatic wheels. However, other forces can affect robot motion while climbing, such as gravity force and friction force. The interaction between the robot and contact surface during climbing can be understood from the balance analysis of several forces, which act in the coupling, as detailed and discussed in Table 1. During a climb path, these forces affect the robot’s mobility differently, according to the surface inclination angle. Figure 1 illustrates the force analysis for three different inclination angles of the contact surface where *v* indicates the robot’s direction of navigation.

At Figure 1, the adhesion intensifies the friction force that is directly proportional to the absolute value of the difference among adhesion and normal forces, which vary according to the wall’s inclination and robot’s position. If the adhesion force is not enough, the contact with the surface may be lost and the robot can fall. On the other hand, if the adhesion force is strong, the required torque on wheels to robot motion will be high. Therefore, the adhesion and normal forces must be balanced to ensure robot adhesion on the surface but also to allow a soft robot motion.

**Figure 2:**
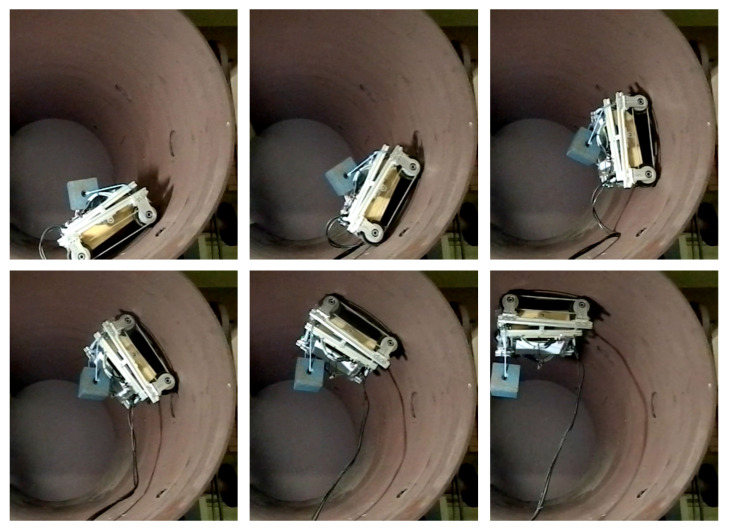
The robot discussed at [22] is able to climb a cylindrical vessel’s inner wall carrying a payload of 12 kg.

Climbing robots are a crucial tool for the automation of inspection processes in the petrochemical industry’s storage tanks. The robot should move around the whole surface while carrying the inspection equipment and not falling when traversing dirty surfaces or when overcoming weld beads. In this situation, the controller cannot neglect the adhesion influences without the risk of falling. An example of a climbing robot for inspection tasks is discussed in [22]. This robot is a four-wheel magnetic differential robot (Figure 1), and it will be used in this paper to validate the proposed control approach. The robot has four misaligned wheels with 43.25 mm of radius, and the tire is made with a polyurethane rubber (with a thickness of 1 mm). These wheels are carefully designed with permanent NdFeB magnets to achieve a maximum adhesion with the correct orientation of magnetic fields, achieving an average adhesion force of 45 kgf on each wheel [22]. The magnetic wheels ensure that the robot supports its weight, inspection equipment, and umbilical cord (power supply) as shown in Figure 2. In this figure, the robot climbs the cylindrical wall of a pressure vessel with a payload of 12 kg.

In general, the papers about climbing robots using dynamic models are usually developed for ground robots. Such models do not consider the interaction between robot and environment, despising some significant effects (as adhesion and gravity) that are crucial to climbing robot modeling. Mobile robots can have their dynamic models expressed by different approaches, but the most common are Lagrange-Euler [29,30,31] and Newton-Euler [32,33,34] based models. The primary difference between these approaches is how to interpret the robot behavior. Newton-Euler approach is based on the comprehension of forces and moments acting over the robot and Lagrange-Euler approach is based on energy analysis, considering specially the effects of kinetic and potential energy on robot’s behavior.

In all above cited works, regardless of the adopted approach (Newton-Euler or Lagrange-Euler), the dynamic analysis is simplified, and crucial features are ignored since the climbing robots are analyzed as a ground robot. However, as discussed, the climbing motion has some fundamental requirements that cannot be neglected during dynamic modeling. An incorrect analysis can weaken the interaction between robot and surface, causing robots to fall and damaging its structure. Thus, the present work aims to contribute to the study of climbing robots, presenting a detailed dynamic model of a wheeled climbing robot considering the friction, gravitational, and adhesion forces. The proposed model considers the interaction between the climbing robot and contact plane, including the main dynamic effects of climb tasks based on the Lagrange-Euler approach. The dynamic and kinematic analysis is developed for a differential four-wheeled climbing robot since this is the most popular topology for this kind of robot.

## 4. Kinematic Analysis

This section brings a 2D kinematic analysis of four-wheeled climbing robots. Kinematic analysis allows us to determine the relative motion of robot wheels to generate a desired global movement. This analysis expresses the robot’s behavior in terms of velocities without any concern about dynamic effects, computing the robot motion in 2D only from its wheel’s analysis. The velocity behavior is very similar for ground and climbing robots. In both cases, the robots do not have Degrees of Freedom (DoF) to perform motion in vertical planes. A climbing robot executes non-ground movements due to its interaction (adhesion system) with a contact plane that lets it track the surface.

**Figure 3:**
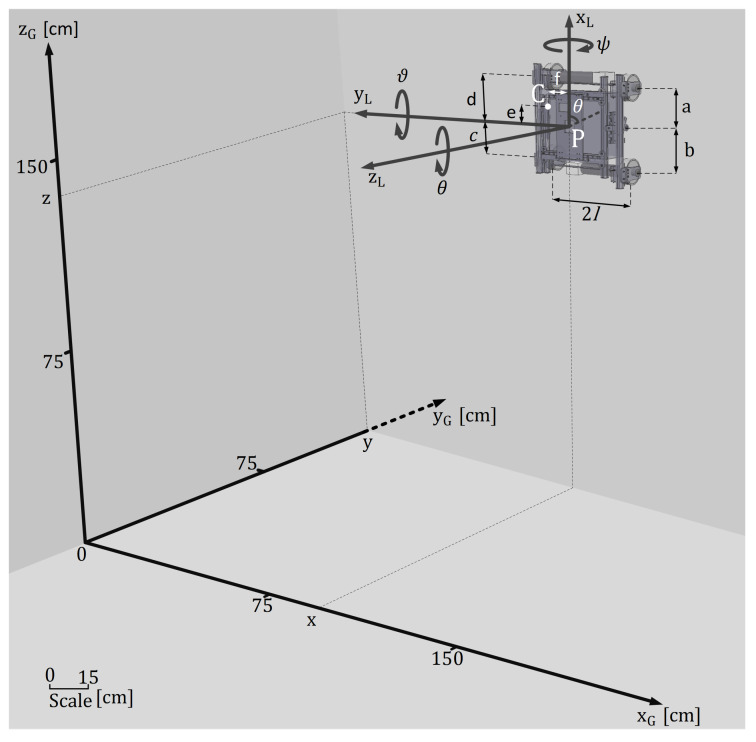
Coordinate frames in differential four-wheeled climbing robot.

Climbing robots are specially designed to achieve optimal adhesion during navigation over the contact plane. Thus, their topology is traditionally more complex compared to ground robots because they have unique features to guarantee a correct interaction with the surface. In this kind of mobile robot, the center of mass does not need to be in the same locus as the geometric center. Another particular characteristic is the alignment of the wheels. In climbing robot design, all wheels can be misaligned to prevent more than one wheel over-passing an obstacle at the same time, hampering a robot fall. Climbing robots’ specific characteristics introduce new requirements in conventional kinematic analysis: wheel misalignment and position of the center of mass, as shown in Figure 3.

The locomotion systems of a climbing robot can perform three-dimensional movements through the interaction between its adhesion systems and the contact plane. Its ability to dynamically interact with the contact plane allows the surface to be tracked, even on non-ground planes. The kinematics of the climbing robot is not influenced by the robot’s spatial movement, but it is highly dependent on the robot’s topological features.

The global coordinate system *G* is the inertial (absolute) reference, and it is fixed on the environment (Figure 3). The local coordinate system *P* corresponds to the robot reference set on its geometric center. The robot’s position (x,y) and orientation (θ) considering the global reference is given by

(1) $$\xi_{G}={\left[\begin{array}{ccc} x & y & \theta \end{array}\right]}^{T}.$$

The relationship between global and local coordinate systems can be defined as

(2) $$\xi_{G}=R_{z}\left(\theta \right)\;\xi_{P},$$

where $\xi_{P}={\left[\begin{array}{ccc} x_{P} & y_{P} & \theta \end{array}\right]}^{T}$ is the robot’s position and orientation in the local reference and $R_{z}\left(\theta \right)$ is the rotation matrix that represents the transformation from robot’s coordinates to the global reference system (around yaw), defined as

(3) $$R_{z}\left(\theta \right)=\left[\begin{array}{ccc} \cos(\theta ) & -\sin(\theta ) & 0\\ \sin(\theta ) & \cos(\theta ) & 0\\ 0 & 0 & 1 \end{array}\right].$$

Mobile robots also require an inverse kinematic analysis allowing the estimation of wheel speeds in terms of desired velocities to the robot. Thus, the angular velocity ($\dot{\phi }$) of the wheel *i* in the local frame can be written as

(4) $${\dot{\phi }}_{i}=\underset{\begin{array}{c} \mathrm{linear}\;\mathrm{motion}\\ \mathrm{in}\;\mathrm{local}\;\mathrm{frame} \end{array}}{\underbrace{\frac{v}{r}}}\pm \;\underset{\begin{array}{c} \mathrm{angular}\;\mathrm{motion}\\ \mathrm{in}\;\mathrm{local}\;\mathrm{frame} \end{array}}{\underbrace{\frac{\omega }{r}∥l_{i}∥}},$$

where, *v* is the linear velocity in the robot frame, *r* is the radius of wheel *i*, ω is the angular velocity in the robot frame, $l_{i}$ is the distance vector between the center of wheel *i* and robot frame, ± represents the direction of motor speed to forward motion, ∥.∥ is the Euclidean norm.

The main topological feature affecting kinematic analysis is the misalignment of the wheels. Thus, inverse kinematics of climbing robots is expressed in terms of robot velocities as

(5) $${\dot{\phi }}_{fr}=\frac{v}{r}+\frac{\omega }{r}\sqrt{a^{2}+l^{2}},$$

(6) $${\dot{\phi }}_{rr}=\frac{v}{r}+\frac{\omega }{r}\sqrt{b^{2}+l^{2}},$$

(7) $${\dot{\phi }}_{rl}=\frac{v}{r}-\frac{\omega }{r}\sqrt{c^{2}+l^{2}},$$

(8) $${\dot{\phi }}_{fl}=\frac{v}{r}-\frac{\omega }{r}\sqrt{d^{2}+l^{2}}.$$

where, *a*, *b*, *c* and *d* are respectively the distance from the center of wheel fr (front right), rr (rear right), rl (rear left) and fl (front left) to the robot’s center (P) on the *y*-axis, and *l* is the distance between the wheels center and the robot’s center (P) on the *x*-axis.

The forward kinematics can be obtained adding (5)–(8) and isolating *v*/ω to express the robot velocities in terms of wheel speeds as

(9) $$v=\frac{r}{4}\left({\dot{\phi }}_{fr}+{\dot{\phi }}_{rr}+{\dot{\phi }}_{rl}+{\dot{\phi }}_{fl}\right),$$

(10) $$\omega =\frac{r}{4}\left(\frac{{\dot{\phi }}_{fr}}{\sqrt{a^{2}+l^{2}}}+\frac{{\dot{\phi }}_{rr}}{\sqrt{b^{2}+l^{2}}}-\frac{{\dot{\phi }}_{rl}}{\sqrt{c^{2}+l^{2}}}-\frac{{\dot{\phi }}_{fl}}{\sqrt{d^{2}+l^{2}}}\right).$$

Wheel speeds should be defined concerning the global coordinate system (or absolute), but in this case, new requirements must be considered. The speed of each wheel can be expressed as

(11) $$v_{w_{i}}=v_{P}+\omega_{P}\times R_{z}\;d_{i},$$

where, $v_{P}$ is the linear velocity of robot, $\omega_{P}$ is the angular velocity of robot, operation × is the vectoring product in terms of generalized coordinates (*x*, *y* and θ) and $d_{i}$ is the distance vector between the wheel’s center *i* and robot’s center of mass *C*. The distance vectors to each wheel are defined as

(12) $$\begin{array}{cccc} d_{fr}=\left[\begin{array}{c} a-e\\ -l+f\\ 0 \end{array}\right], & d_{rr}=\left[\begin{array}{c} -b+e\\ -l+f\\ 0 \end{array}\right], & d_{rl}=\left[\begin{array}{c} -c+e\\ l-f\\ 0 \end{array}\right], & d_{fl}=\left[\begin{array}{c} d-e\\ l-f\\ 0 \end{array}\right] \end{array}.$$

where, *f* is the distance between the center of mass (C) and the robot’s center (P) on the *y*-axis, and *e* is the distance between the center of mass and the robot’s center on the *x*-axis.

The wheel speeds can be similarly written applying the Equation (11) and using the distance vectors at Equation (12), resulting in

(13) $$v_{w_{fr}}=\left[\begin{array}{c} \dot{x}+a\dot{\theta }\cos\left(\theta \right)-e\dot{\theta }\cos\left(\theta \right)+l\dot{\theta }\sin\left(\theta \right)-f\dot{\theta }\sin\left(\theta \right)\\ \dot{y}+a\dot{\theta }\sin\left(\theta \right)-e\dot{\theta }\sin\left(\theta \right)-l\dot{\theta }\cos\left(\theta \right)+f\dot{\theta }\cos\left(\theta \right)\\ 0 \end{array}\right],$$

(14) $$v_{w_{rr}}=\left[\begin{array}{c} \dot{x}-b\dot{\theta }\cos\left(\theta \right)+e\dot{\theta }\cos\left(\theta \right)+l\dot{\theta }\sin\left(\theta \right)-f\dot{\theta }\sin\left(\theta \right)\\ \dot{y}-b\dot{\theta }\sin\left(\theta \right)+e\dot{\theta }\sin\left(\theta \right)-l\dot{\theta }\cos\left(\theta \right)+f\dot{\theta }\cos\left(\theta \right)\\ 0 \end{array}\right],$$

(15) $$v_{w_{rl}}=\left[\begin{array}{c} \dot{x}-c\dot{\theta }\cos\left(\theta \right)+e\dot{\theta }\cos\left(\theta \right)-l\dot{\theta }\sin\left(\theta \right)+f\dot{\theta }\sin\left(\theta \right)\\ \dot{y}-c\dot{\theta }\sin\left(\theta \right)+e\dot{\theta }\sin\left(\theta \right)+l\dot{\theta }\cos\left(\theta \right)-f\dot{\theta }\cos\left(\theta \right)\\ 0 \end{array}\right],$$

(16) $$v_{w_{fl}}=\left[\begin{array}{c} \dot{x}+d\dot{\theta }\cos\left(\theta \right)-e\dot{\theta }\cos\left(\theta \right)-l\dot{\theta }\sin\left(\theta \right)+f\dot{\theta }\sin\left(\theta \right)\\ \dot{y}+d\dot{\theta }\sin\left(\theta \right)-e\dot{\theta }\sin\left(\theta \right)+l\dot{\theta }\cos\left(\theta \right)-f\dot{\theta }\cos\left(\theta \right)\\ 0 \end{array}\right].$$

The differential four-wheeled robot is a nonholonomic system, since the constraint imposed on its kinematic model is non-integrable. The robot’s wheels must have pure rolling and non-slipping characteristics. The lateral slip constraint defines that the robot cannot have lateral motion, that is, the robot linear velocity along the *y*-axis is null (${\dot{y}}_{P}=0$). Thus, non-slipping constraint is defined as

(17) $$-\dot{x}\sin\left(\theta \right)+\dot{y}\cos\left(\theta \right)=0.$$

The pure rolling constraint implies that the velocity of the center of the wheel is proportional to the angular velocity of the wheel. The pure rolling constraint of a wheeled robot can be expressed as

(18) $${\dot{x}}_{w_{i}}\cos\left(\theta \right)+{\dot{y}}_{w_{i}}\sin\left(\theta \right)=r{\dot{\phi }}_{i},$$

where, ${\dot{x}}_{w_{i}}$ is the linear velocity along the *x*-axis of wheel *i* and ${\dot{y}}_{w_{i}}$ is the linear velocity along the *y*-axis of wheel *i*.

The kinematic constraints matrix of non-ground four-wheeled robots are obtained applying the non-slipping constraints (Equation (17)) and pure rolling constraints (Equation (18)) in equations of wheel speeds (Equations (13)–(16)) resulting in

(19) $$A\left(q\right)=\left[\begin{array}{ccccccc} -\sin(\theta ) & \cos(\theta ) & 0 & 0 & 0 & 0 & 0\\ \cos(\theta ) & \sin(\theta ) & a-e & -r & 0 & 0 & 0\\ \cos(\theta ) & \sin(\theta ) & -b+e & 0 & -r & 0 & 0\\ \cos(\theta ) & \sin(\theta ) & -c+e & 0 & 0 & -r & 0\\ \cos(\theta ) & \sin(\theta ) & d-e & 0 & 0 & 0 & -r \end{array}\right].$$

From Equation (19), the kinematic constraints matrix A(q) is composed of the non-slipping and rolling restrictions. The first line of A(q) matrix is the non-slipping constraint, and the subsequent lines are the pure rolling constraint of each robot’s wheel.

The above kinematic analysis of a non-ground robot allows us to define the robot’s speed from the wheels. Also, the reference of the robot’s velocity can be changed according to its specifications. This section presented the kinematic model (Equations (6) to (19)), and it can be used in the most diverse climbing robots. The next step is to perform a dynamic analysis to understand how forces act in the robot’s motion.

## 5. Dynamic Analysis

Solid knowledge about the robot’s dynamic characteristics is a fundamental requirement to successfully implement control tasks, e.g., path following and trajectory tracking. Dynamic analysis aims to describe the physical behavior of the robot considering internal (i.e., the synergy between its elements) and external (i.e., the interaction between the robot and environment) aspects. This analysis expresses the robot as a set of rigid bodies that applies forces and torques in another system elements, differing from kinematic analysis that considers only geometric aspects.

Climbing robots can execute motions in planes orthogonal to the ground due to their interaction (adhesion system) with the environment, as shown in Figure 1. In this context, it is impossible to ignore or disregard some dynamic influences like the adhesion system. The interaction with the surface introduces a dynamic behavior that is dependent on the robot orientation. Thus, gravitational effect cannot be despised, and this interaction must be included in any model that is closer to reality.

Lagrange models are easy to derive and result in scalar equations, circumventing the knowledge about constraint forces and also avoiding acceleration computation needed to Newton-Euler formulation. Thus, this approach is adopted in this paper to derive the dynamic model of a climbing robot.

The classical dynamic equation for mobile robots can be expressed, according to [35], as

(20) $$M\left(q\right)\ddot{q}+V\left(q,\dot{q}\right)\dot{q}+E\left(\dot{q}\right)+G\left(\varphi \right)+\tau_{d}=B\left(q\right)\tau -A^{T}\left(q\right)\lambda ,$$

where, *M* is the symmetric inertial matrix, *V* is the centripetal and Coriolis matrix, *E* is the interaction matrix between surface and robot including friction effects, *G* is the gravitational vector, φ is the robot’s spatial orientation vector defined as $\varphi ={\left[\begin{array}{ccc} \theta & \vartheta & \psi \end{array}\right]}^{T}$, $\tau_{d}$ is the vector of bounded unknown disturbances including unmodeled dynamics, *B* is the input matrix, τ is the input vector, *A* is the matrix associated with kinematic constraints, λ is the vector of Lagrange multipliers, *q* is the generalized coordinates defined as q = ${\left[\begin{array}{ccccccc} x & y & \theta & \phi_{1} & \phi_{2} & \phi_{3} & \phi_{4} \end{array}\right]}^{T}$.

As discussed, the robot dynamic modeling based on the Lagrange-Euler approach is carried out by analyzing the kinetic and potential energy of the system. The Lagrange-Euler equation is expressed as

(21) $$\frac{d}{dt}\left(\frac{\partial L}{\partial \dot{q}}\right)-\frac{\partial L}{\partial q}=Q-A^{T}\left(q\right)\lambda ,$$

where, L=K−U is the difference between the kinetic energy (K) and potential energy (U), *Q* is the generalized force vector, *A* is the matrix associated with kinematic constraints, and λ is the vector of Lagrange multipliers.

In climbing robots, the potential energy (U) can be disregarded in Equation (21) due to the robot’s adhesion system. So, the total kinetic energy of the system is the sum of the kinetic energy of the wheeled robot despising the wheels and the kinetic energy of the wheels with actuators. Using Köenig’s theorem [36], the kinetic energy of the robot without considering the wheels is

(22) $$K_{P}=\frac{1}{2}m_{P}\left({\dot{x}}_{P}^{2}+{\dot{y}}_{P}^{2}\right)+\frac{1}{2}I_{P}{\dot{\theta }}^{2},$$

where, $m_{P}$ is the robot’s mass that despises wheels and actuators and $I_{P}$ is the moment of inertia of the robot. Moreover, the kinetic energy of the wheels can be defined as

(23) $$\begin{array}{cc} K_{w_{fr}}=\frac{1}{2}m_{w}v_{w_{fr}}^{2}+\frac{1}{2}I_{m}{\dot{\theta }}^{2}+\frac{1}{2}I_{w}{\dot{\phi }}_{fr}^{2},\; & K_{w_{rr}}=\frac{1}{2}m_{w}v_{w_{rr}}^{2}+\frac{1}{2}I_{m}{\dot{\theta }}^{2}+\frac{1}{2}I_{w}{\dot{\phi }}_{rr}^{2},\\ K_{w_{rl}}=\frac{1}{2}m_{w}v_{w_{rl}}^{2}+\frac{1}{2}I_{m}{\dot{\theta }}^{2}+\frac{1}{2}I_{w}{\dot{\phi }}_{rl}^{2},\; & K_{w_{fl}}=\frac{1}{2}m_{w}v_{w_{fl}}^{2}+\frac{1}{2}I_{m}{\dot{\theta }}^{2}+\frac{1}{2}I_{w}{\dot{\phi }}_{fl}^{2}, \end{array}$$

where, $m_{w}$ is the mass of the wheel with the actuator, $v_{w_{i}}$ is the wheel velocity, $I_{m}$ is the moment of inertia of the wheel with actuator about the wheel diameter and $I_{w}$ is the moment of inertia of the wheel with actuator about the wheel axis.

The total kinetic energy can be written as

(24) $$\begin{array}{c} L=K_{T}=\frac{1}{2}m\left({\dot{x}}^{2}+{\dot{y}}^{2}\right)+m_{P}\dot{\theta }\left[\dot{y}\left(e\cos(\theta )-f\sin(\theta )\right)-\dot{x}\left(e\sin(\theta )+f\cos(\theta )\right)\right]\\ +m_{w}\dot{\theta }\left(a-b-c+d\right)\left(\dot{y}\cos\left(\theta \right)-\dot{x}\sin\left(\theta \right)\right)+\frac{1}{2}I{\dot{\theta }}^{2}+\frac{1}{2}I_{w}\left({\dot{\phi }}_{fr}^{2}+{\dot{\phi }}_{rr}^{2}+{\dot{\phi }}_{rl}^{2}+{\dot{\phi }}_{fl}^{2}\right) \end{array}$$

where, $m=m_{P}+4m_{w}$ is the total mass of the climbing robot, and I is the total inertia represented as

(25) $$\begin{array}{c} I=I_{P}+4I_{m}+m_{w}\left(a^{2}+b^{2}+c^{2}+d^{2}\right)+4m_{w}\left(l^{2}-2lf\right)+m\left(e^{2}+f^{2}\right)-2m_{w}e\left(a+b+c+d\right) \end{array}$$

Introducing the total kinetic energy Equation (24) into the Lagrange-Euler Equation (21), the robot’s motion matrices are presented in the Appendix A.

The solution of Lagrange multipliers in Equation (21) is not a trivial task, so to eliminate the kinematic constraints matrix A(q), $\dot{q}$ is rewritten as

(26) $$\dot{q}=S\left(q\right)\eta ,$$

where, $\eta ={\left[\begin{array}{cccc} {\dot{\phi }}_{fr} & {\dot{\phi }}_{rr} & {\dot{\phi }}_{rl} & {\dot{\phi }}_{fl} \end{array}\right]}^{T}$, S(q) is defined as

(27) $$\begin{array}{c} S\left(q\right)=\left[\begin{array}{cccc} \frac{r}{4}\cos\left(\theta \right) & \frac{r}{4}\cos\left(\theta \right) & \frac{r}{4}\cos\left(\theta \right) & \frac{r}{4}\cos\left(\theta \right)\\ \frac{r}{4}\sin\left(\theta \right) & \frac{r}{4}\sin\left(\theta \right) & \frac{r}{4}\sin\left(\theta \right) & \frac{r}{4}\sin\left(\theta \right)\\ \frac{r}{4\sqrt{a^{2}+l^{2}}} & \frac{r}{4\sqrt{b^{2}+l^{2}}} & \frac{-r}{4\sqrt{c^{2}+l^{2}}} & \frac{-r}{4\sqrt{d^{2}+l^{2}}}\\ 1 & 0 & 0 & 0\\ 0 & 1 & 0 & 0\\ 0 & 0 & 1 & 0\\ 0 & 0 & 0 & 1 \end{array}\right] \end{array}$$

Thus, the time derivative of Equation (26) results in

(28) $$\ddot{q}=S\left(q\right)\dot{\eta }+\dot{S}\left(q\right)\eta .$$

Therefore, Equations (26)–(28) can be introduced into Equation (20) and be multiplied by transpose of S(q), resulting in

(29) $$\begin{array}{c} S^{T}\left(q\right)M\left(q\right)\left[S\left(q\right)\dot{\eta }+\dot{S}\left(q\right)\eta \right]+S^{T}\left(q\right)V\left(q,\dot{q}\right)S\left(q\right)\eta \\ +S^{T}\left(q\right)E\left(\dot{q}\right)+S^{T}\left(q\right)G\left(\phi \right)+S^{T}\left(q\right)\tau_{d}\\ =S^{T}\left(q\right)B\left(q\right)\tau -S^{T}\left(q\right)A^{T}\left(q\right)\lambda , \end{array}$$

As S(q) is null space of the kinematic constraints matrix A(q), i.e., $S^{T}\left(q\right)A^{T}\left(q\right)\lambda =0$, Equation (29) is simplified to

(30) $$\begin{array}{c} S^{T}\left(q\right)M\left(q\right)S\left(q\right)\dot{\eta }+S^{T}\left(q\right)\left[M\left(q\right)\dot{S}\left(q\right)+V\left(q,\dot{q}\right)S\left(q\right)\right]\eta \\ +S^{T}\left(q\right)F\left(\dot{q}\right)+S^{T}\left(q\right)G\left(q\right)+S^{T}\left(q\right)\tau_{d}=S^{T}\left(q\right)B\left(q\right)\tau . \end{array}$$

As a result, new matrices of the dynamic model can be defined as

(31) $$\begin{array}{c} \bar{M}\left(q\right)=S^{T}\left(q\right)M\left(q\right)S\left(q\right)\\ \bar{V}\left(q,\dot{q}\right)=S^{T}\left(q\right)\left[M\left(q\right)\dot{S}\left(q\right)+V\left(q,\dot{q}\right)S\left(q\right)\right]\\ \bar{E}\left(\dot{q}\right)=S^{T}\left(q\right)E\left(\dot{q}\right)\\ \bar{G}\left(q\right)=S^{T}\left(q\right)G\left(q\right)\\ {\bar{\tau }}_{d}=S^{T}\left(q\right)\tau_{d}\\ \bar{B}\left(q\right)=S^{T}\left(q\right)B\left(q\right) \end{array}$$

Comparing Equations (20) and (30) and using the variables defined at Equation (31), the classic dynamic of a rigid body can be rewritten without Lagrange multipliers, as

(32) $$\bar{M}\left(q\right)\dot{\eta }+\bar{V}\left(q,\dot{q}\right)\eta +\bar{E}\left(\dot{q}\right)+\bar{G}\left(q\right)+{\bar{\tau }}_{d}=\bar{B}\left(q\right)\tau .$$

The contribution of gravity and interaction forces must be included in the dynamic model of the climbing robot. In the next sections, the detailed analysis of these influences will be presented.

### 5.1. Gravity Model

The gravity force is dependent on the spatial orientation of the robot body during its navigation. Robot’s spatial orientation is defined by the angles of roll (angle on the *x*-axis), pitch (angle on the *y*-axis) and yaw (angle on the *z*-axis), resulting in

(33) $$G\left(\varphi \right)={\left[\begin{array}{cccccc} -mg\sin(\vartheta & m\cos(\vartheta )\sin(\psi ) & 0 & 0 & 0 & 0 \end{array}\right]}^{T}$$

where, *m* is the total mass of the climbing robot, φ is robot’s spatial orientation vector, ψ is the roll angle, ϑ is the pitch angle, θ is the yaw angle, and *g* is the value of gravity acceleration vector (g=−9.81 m/s${}^{2}$).

The gravitational influence can be rewritten in terms of $\bar{G}\left(\varphi \right)$, as presented in Equation (31), resulting in

(34) $$G\left(\varphi \right)=\left[\begin{array}{c} mgr\left(\frac{\cos(\vartheta )sin(\psi )\sin(\theta )-\cos(\theta )sin(\vartheta )}{4}\right)\\ mgr\left(\frac{\cos(\vartheta )sin(\psi )\sin(\theta )-\cos(\theta )sin(\vartheta )}{4}\right)\\ mgr\left(\frac{\cos(\vartheta )sin(\psi )\sin(\theta )-\cos(\theta )sin(\vartheta )}{4}\right)\\ mgr\left(\frac{\cos(\vartheta )sin(\psi )\sin(\theta )-\cos(\theta )sin(\vartheta )}{4}\right) \end{array}\right]$$

### 5.2. Interaction Model

The interaction is the primordial influence of climbing robots, which cannot be ignored with the risk of damages. This factor is time-variant and describes the coupling of wheels friction and adhesion force, as illustrated in Figure 1. Moreover, the normal force on the robot’s wheels is a reaction of the robot’s compression against the surface, which strongly influences the friction force. Various elements can influence the interaction force, such as the material of the wheels and the contact surface, because this affects the friction coefficient, causing the force to increase or not its value. Thus, the interaction model depends on the contact between surface and adhesion element, as defined in Equation (35).

(35) $$\begin{array}{cc} E_{x_{i}}=\mu_{x}N_{w_{i}}sgn\left({\dot{x}}_{w_{i}}\right), & E_{y_{i}}=\mu_{y}N_{w_{i}}sgn\left({\dot{y}}_{w_{i}}\right), \end{array}$$

where, $E_{x_{i}}$ is the interaction force by the rolling motion, $E_{y_{i}}$ is the interaction force by the sliding motion, $\mu_{x}$ is the rolling resistive coefficient, $\mu_{y}$ is the sliding resistive coefficient, $N_{w_{i}}$ is the normal force on wheel *i* due to compression of gravity and adhesion forces, ${\dot{x}}_{w_{i}}$ is the linear velocity on the *x*-axis of the wheel *i*, ${\dot{y}}_{w_{i}}$ is the linear velocity on the *y*-axis of the wheel *i*.

Normal forces of climbing robot wheels can be described as

(36) $$N_{w}=\frac{∥d_{i}∥}{∥d_{fr}∥+∥d_{rr}∥+∥d_{rl}∥+∥d_{fl}∥}m∥g∥\cos\left(\psi \right)\cos\left(\vartheta \right)+∥F_{ad}∥,$$

where, *w* is robot’s wheel (fr = front-right, fl = front-left, rr = rear-right, rl = rear-left), $∥d_{i}∥$ is the Euclidean distance between the wheel’s center *i* and the robot’s center of gravity *C* and $∥F_{ad}∥$ is the absolute value of the adhesion force of each wheel in Newton.

The adhesion force of the robot’s wheels $F_{ad}$ is the absolute value of the adhesion method, and its value is dependent on the basic principles (i.e., magnetic or electrostatic) used to robot coupling.

Interaction forces on the *x*-axis and *y*-axis can be expressed in terms of Equations (35) and (36), as

(37) $$\begin{array}{c} E_{x}=E_{x_{fr}}+E_{x_{rr}}+E_{x_{rl}}+E_{x_{rl}}\\ E_{y}=E_{y_{fr}}+E_{y_{rr}}+E_{y_{rl}}+E_{y_{rl}} \end{array}$$

The resistive moment $M_{r}$ is written as

(38) $$\begin{array}{c} M_{r}=\left(a-e\right)E_{y_{fr}}+\left(-b+e\right)E_{y_{rr}}+\left(-c+e\right)E_{y_{rl}}+\left(d-e\right)E_{y_{fl}}\\ +\left(l+f\right)E_{x_{fr}}+\left(l+f\right)E_{x_{rr}}+\left(-l+f\right)E_{x_{rl}}+\left(-l+f\right)E_{x_{fl}} \end{array}$$

The interaction force matrix *E*, which describes the relation of climbing robot and contact surface, can be defined as

(39) $$E\left(\dot{q}\right)={\left[\begin{array}{ccccccc} E_{x} & E_{y} & M_{r} & 0 & 0 & 0 & 0 \end{array}\right]}^{T},$$

and can be rewritten using the Equation (31), resulting in

(40) $$\begin{array}{c} \bar{E}\left(\dot{q}\right)=\left[\begin{array}{c} \frac{E_{x}r\cos\left(\theta \right)}{4}+\frac{E_{y}r\sin\left(\theta \right)}{4}+\frac{M_{r}r}{4\sqrt{a^{2}+l^{2}}}\\ \frac{E_{x}r\cos\left(\theta \right)}{4}+\frac{E_{y}r\sin\left(\theta \right)}{4}+\frac{M_{r}r}{4\sqrt{b^{2}+l^{2}}}\\ \frac{E_{x}r\cos\left(\theta \right)}{4}+\frac{E_{y}r\sin\left(\theta \right)}{4}-\frac{M_{r}r}{4\sqrt{c^{2}+l^{2}}}\\ \frac{E_{x}r\cos\left(\theta \right)}{4}+\frac{E_{y}r\sin\left(\theta \right)}{4}-\frac{M_{r}r}{4\sqrt{d^{2}+l^{2}}} \end{array}\right] \end{array}.$$

These above presented kinematic and dynamic models of a climbing robot are used in this paper to develop a new MPC strategy for velocity tracking based on torque control in robotics.

## 6. Velocity and Torque Control by MPC Approach

One of the most used multi-variable control techniques is the model predictive control, in which the main objective is to minimize/maximize an objective function related to a performance criterion. In general, a good performance of a MPC strategy is related to how close the model is to the system to be controlled. On the other hand, the velocity error minimization during the robot’s navigation is a well known strategy for trajectory tracking problems in robotics. Thus, a model-based controller (MPC) will be used in this paper to perform torque control for a climbing robot’s velocity tracking. The use of MPC control allows minimizing the effects of the nonlinearities present in the climbing robot.

**Figure 4:**
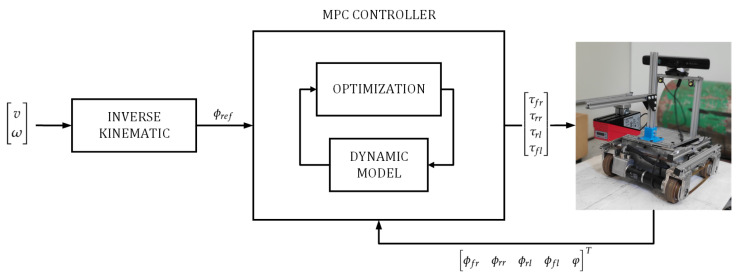
Model-based controller (MPC) diagram.

The proposed MPC controller diagram is shown in Figure 4. The linear and angular speeds are converted into the wheel speeds through the inverse kinematic model and they are input as reference signal to MPC controller ($\phi_{ref}$). In its turn, the MPC controller uses the above developed dynamic model to compensate and control the torque ($\tau ={\left[\begin{array}{cccc} \tau_{fr} & \tau_{fr} & \tau_{fr} & \tau_{fr} \end{array}\right]}^{T}$) of the climbing robot’s wheels for velocity tracking.

The MPC approach is based on a state-space model to design control law. Therefore the dynamic model developed in Section 5 is rewritten in state-space, including an integrator action to attenuate unmodelled errors and disturbance effects. For this, an integrator state is introduced:

(41) $$x_{i}\left(t+1\right)=x_{i}\left(t\right)+\left(ref\left(t\right)-y\left(t\right)\right)$$

where, ref is the reference and y(t) is the system’s output.

The acceleration and velocity are provided as inputs to the dynamic model. Still, since the climbing robot’s purpose is to perform the inspection, only the speed is considered a reference. During the inspection, the robot must navigate at a constant speed. Therefore, the acceleration and deceleration control is disregarded. It will be implicit in the modeling. The state variables are defined such that $x_{\dot{\phi }}={\left[\begin{array}{cccc} {\dot{\phi }}_{fr} & {\dot{\phi }}_{rr} & {\dot{\phi }}_{rl} & {\dot{\phi }}_{fl} \end{array}\right]}^{T}$ is the wheel speeds and $x_{i}$ is the error of the wheel speeds. These two states are the outputs of the MPC controller, and the state-space with integrator equations are defined as:

(42) $$\begin{array}{c} \begin{array}{c} x_{e}\left(t+1\right)=\left[\begin{array}{c} x_{\dot{\phi }}\left(t+1\right)\\ x_{i}\left(t+1\right) \end{array}\right]=\underset{A_{a}}{\underbrace{\left[\begin{array}{cc} A_{ss} & 0\\ -C_{ss} & 0 \end{array}\right]}}x_{e}\left(t\right) \end{array}\underset{B_{a}}{\underbrace{\left[\begin{array}{c} B_{ss}\\ 0 \end{array}\right]}}\underset{K_{a}}{\underbrace{\left[\begin{array}{cc} K_{p} & K_{i} \end{array}\right]}}u_{ss}\left(t\right)+\left[\begin{array}{c} 0\\ 1 \end{array}\right]ref\left(t\right) \end{array}$$

where, the input $u_{ss}$ of state-space of the MPC controller is defined as:

(43) $$u_{ss}\left(t\right)=\bar{M}{\left(q\right)}^{-1}\left(\bar{B}\left(q\right)\tau -\bar{E}\left(\dot{q}\right)-\bar{G}\left(\phi \right)\right)$$

Simplifying the Equation (42):

(44) $$x_{e}\left(t+1\right)=\left(A_{a}-B_{a}K_{a}\right)x_{e}\left(t\right)+\left[\begin{array}{c} 0\\ 1 \end{array}\right]ref\left(t\right)$$

(45) $$y\left(t\right)=C_{ss}x_{e}\left(t\right)$$

where, $A_{ss}$, $B_{ss}$, $C_{ss}$ are detailed in the Appendix B.

From the Equation (43), which defines the manipulated variable of the predictive controller, the input of the robot is controlled through the torque:

(46) $$\tau ={\left[\begin{array}{cccc} \tau_{fr} & \tau_{rr} & \tau_{rl} & \tau_{fl} \end{array}\right]}^{T}=\bar{B}{\left(q\right)}^{-1}\left(\bar{M}\left(q\right)u_{ss}+\bar{E}\left(\dot{q}\right)+\bar{G}\left(\phi \right)\right)$$

The control optimization problem is defined as

(47) $$u_{ss}=\arg\min J$$

The mathematical formulation of the MPC controller is given by [9]:

(48) $$J=\sum_{j=0}^{N_{p}}\delta \left(j\right){\left[\hat{y}\left(t+j|k\right)-ref\left(t+j\right)\right]}^{2}+\sum_{i=1}^{N_{u}}\lambda \left(i\right){\left[\Delta u_{ss}\left(t+i|k\right)\right]}^{2}$$

where, $N_{p}$ is prediction horizon, $N_{u}$ is control horizon, $\hat{y}$ is predicted output of state space model, ref is reference, $\Delta u_{ss}$ is control effort, δ and λ are weighting factors.

## 7. Simulation Results

In this section, the proposed model-based control approach is applied to the trajectory tracking problem of a climbing robot through simulations. First, both developed kinematic and dynamic models are validated by climbing robot performance in three different situations. Since the models are valid, the MPC controller is also essayed and validated using the simulator V-REP.

### 7.1. Analysis of Kinematic and Dynamic Models

An analysis of the torque behavior is carried out using three different simulated scenarios. These simulations allow to verify the dynamic and kinematic models’ performance during climbing over three surfaces with different inclination angles. In the first scenario, the robot navigates on a flat surface (0 degrees) from the point [0,0] to the point [5,0] during 10 s, maintaining its orientation. The second scenario describes the navigation of the climbing robot straight from the ground up on an orthogonal surface (90 degrees), performing the same displacement of the previous experiment. In the last scenario, the climbing robot starts its motion over a flat surface that increases its angle until becomes perpendicular to the ground plane (like in the interior of a sphere).

The robot trajectory planning is based on the algorithm proposed in [37]. The planned values of acceleration and velocity are used as inputs to the dynamic model. Simulation parameters for these three scenarios are presented in Table 2. The robot’s parameters are taken from the real robot shown in Figure 1 and Figure 2. This robot is also used in the real experiments to be presented in Section 8.

From dynamic analysis in Section 5, the computed torque of the climbing robot is given by Equation (49). At the same time, the velocity is obtained by the numerical integration of the acceleration (Equation (50)) using the Euler method.

(49) $$\tau =\bar{B}{\left(q\right)}^{-1}\left(\bar{M}\left(q\right)\dot{\eta }+\bar{V}\left(q,\dot{q}\right)\eta +\bar{E}\left(\dot{q}\right)+\bar{G}\left(\phi \right)\right)$$

(50) $$\dot{\eta }=\bar{M}{\left(q\right)}^{-1}\left(\bar{B}\left(q\right)\tau -\bar{V}\left(q,\dot{q}\right)\eta -\bar{E}\left(\dot{q}\right)-\bar{G}\left(\phi \right)\right)$$

**Figure 5:**
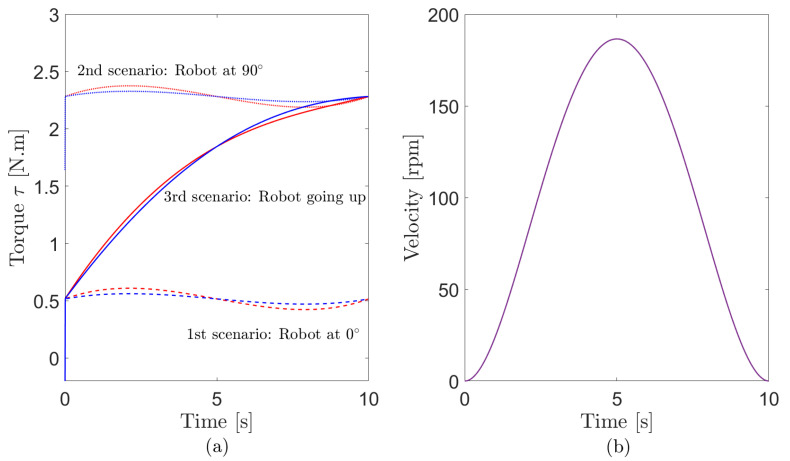
Simulation results of Kinematic and Dynamic models: right wheel torque (red) and left wheel torque (blue): red(**a**) Torque output in simulation. (**b**) Velocity profile in simulation.

The results are shown in Figure 5, where the torque and velocity of the robot are displayed for the three scenarios. In Figure 5a, the torque required for the robot’s movement is quite different on each of the scenarios. However, the three situations have the same speed profile, so the robot performs the same trajectory. Figure 5b testifies that the climbing robot speed profile is the same in the three situations, proving that they showed the same displacement.

As expected, the robot needs a lower torque when on the flat surface than when climbing during its motion in the first scenario, since when on a flat surface, the effects of gravity and interaction force are smaller. In particular, for the third scenario, the robot required torque is growing because, at the beginning of the climb (3rd scenario), the interaction force is higher due to the sum of the reactive force to the weight and adhesion forces of the wheel. For the second situation, where the robot is already at 90 degrees, the normal force is composed only by the adhesion force, which decreases the interaction force. Thus the required torque is higher, but its profile is similar to the torque from the first scenario.

### 7.2. The Proposed MPC Controller Validation

**Figure 6:**
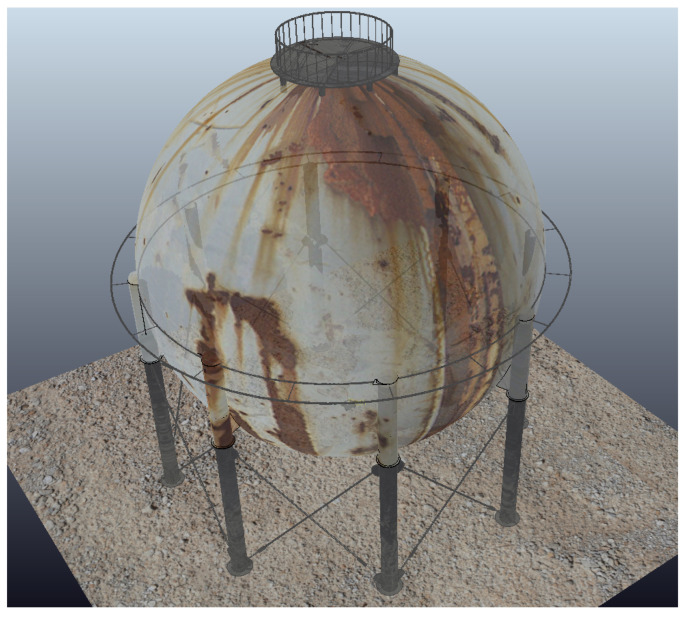
Virtual spherical tank in the V-REP simulator.

**Figure 7:**
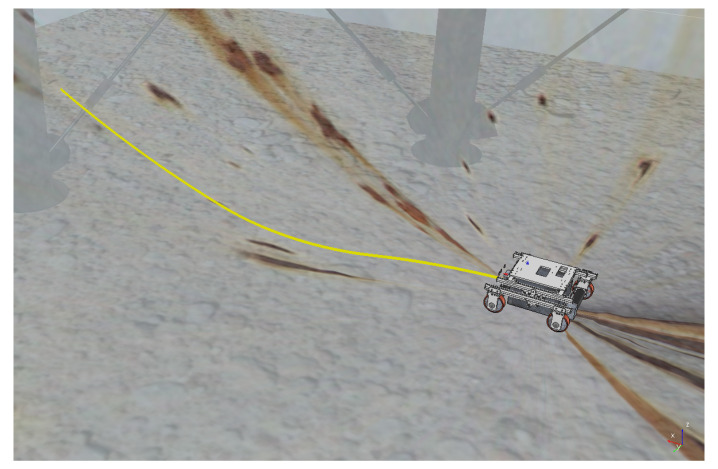
Trajectory of the climbing robot during navigation in the storage tank.

The experiments to validate the MPC controller are simulated inside a virtual LPG storage spherical pressure vessel developed in the virtual experimentation platform V-REP, as shown in Figure 6. V-REP is a 3D robot simulator based on a distributed control architecture allowing the user to run MATLAB code, where MPC controller is implemented. The V-REP dynamics module simulates interactions between the robot and others objects in a very similar way to the real world. Because the surface of the spherical tank is concave, presenting variations in the slopes, this environment constitutes a good test-bed to verify the robustness of the proposed MPC controller under different dynamic effects. The climbing robot is modeled on the V-REP retaining the same characteristics as the real robot, such as weight and adhesion system. The entire structure of the MPC controller was designed in a MATLAB script. The robot’s trajectory inside the spherical tank is shown in Figure 7 with different speed references.

The parameters and limits used to tune the MPC controller are shown in Table 3.

**Figure 8:**
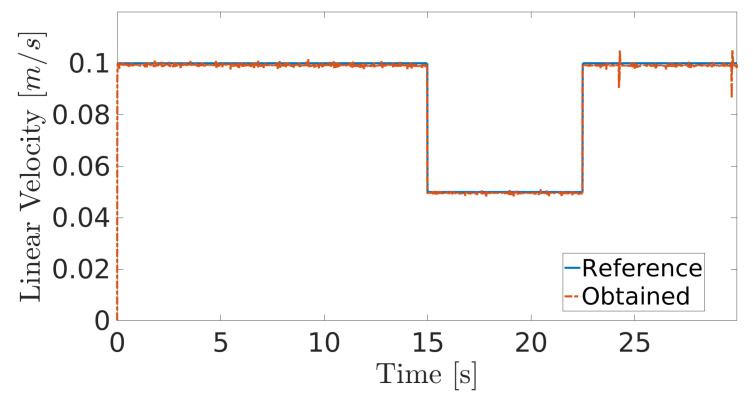
Linear velocity during navigation of the climbing robot in the storage tank.

**Figure 9:**
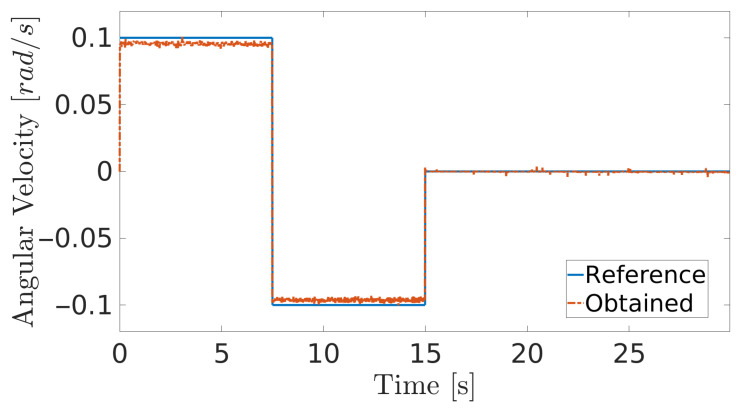
Angular velocity during navigation of the climbing robot in the storage tank.

**Figure 10:**
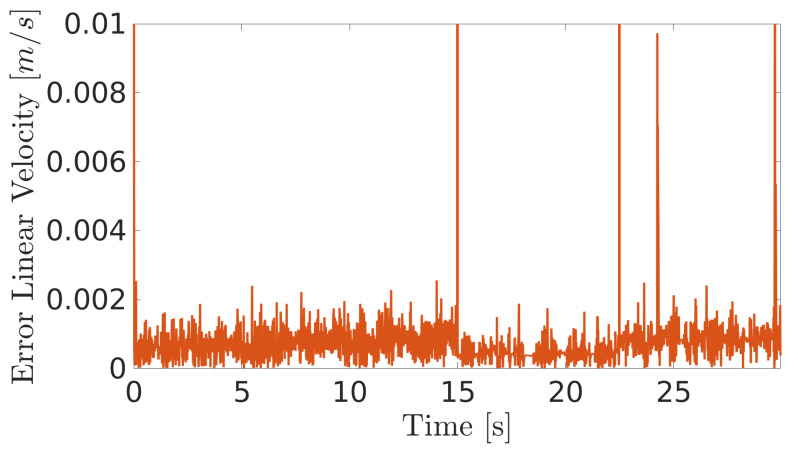
Linear velocity error during navigation of the climbing robot in the storage tank.

**Figure 11:**
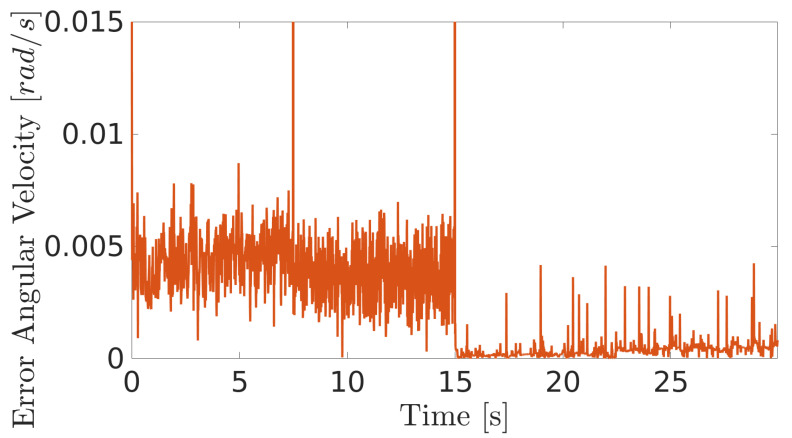
Angular velocity error during navigation of the climbing robot in the storage tank.

The obtained results are given in Figure 8 and Figure 9. The MPC controller achieves an insignificant error for the reference velocities, as shown in Figure 10 and Figure 11. In Figure 8, there are two peaks in the linear speed. They are due to some defective points in the virtual simulated tank model, which ends up generating a variation in the dynamics of the robot. But despite this, the proposed controller proves to be robust, since it quickly rejects these disturbances that may happen, and soon restores the speed tracking generating an insignificant error.

**Figure 12:**
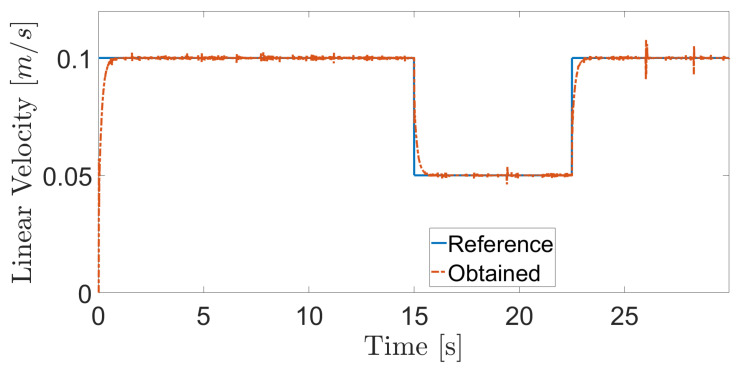
Linear velocity in the PI controller during navigation of the climbing robot in the storage tank.

**Figure 13:**
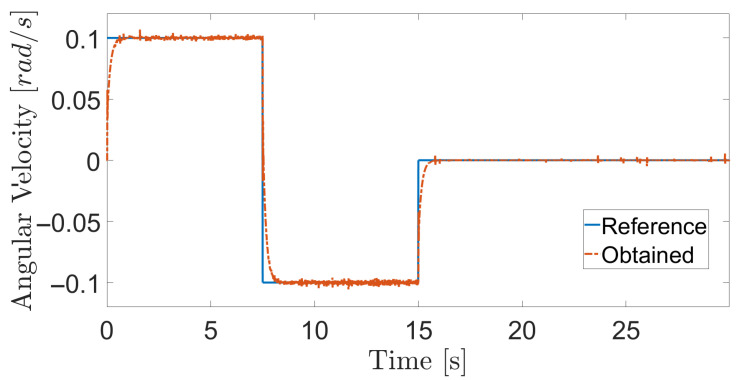
Angular velocity in the PI controller during navigation of the climbing robot in the storage tank.

A classical PI controller is also designed allowing a performance comparison with the proposed controller. The PI controller’s performance for the speed tracking of the inspection robot is shown in Figure 12 and Figure 13. Note that the PI response is slower when compared to the proposed MPC controller. This slower time response causes a more significant variation in the robot’s inspection speed, which is not suitable. Inspection tasks require that the robot must move at a constant velocity. Anticipation actions is one of the advantages of the MPC over other control strategies, making the proposed controller’s response faster. Also, the MPC controller has better disturb rejection than the PI controller.

**Figure 14:**
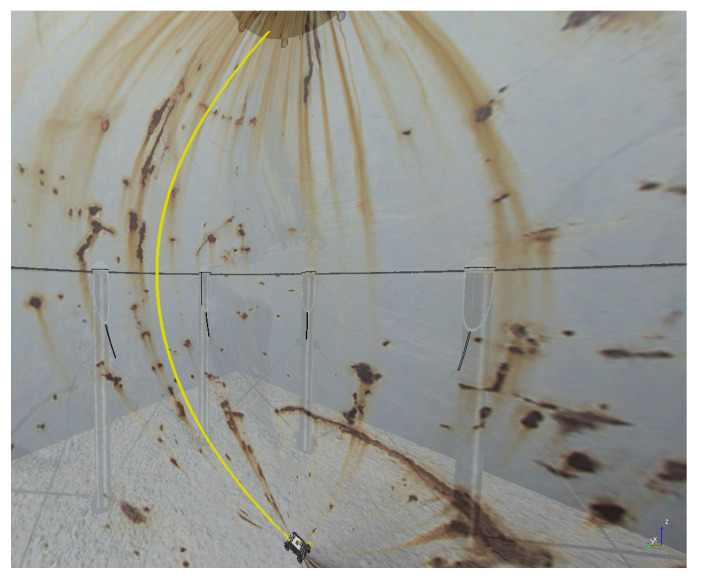
Robot trajectory while climbing the LPG tank.

**Figure 15:**
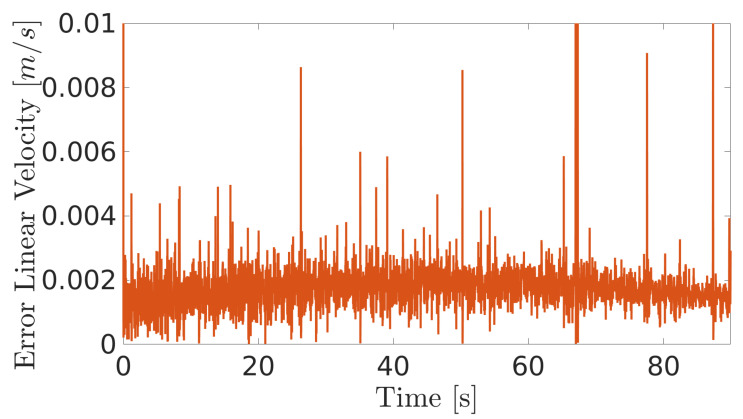
Linear velocity error of the robot when climbing the inner surface of the LPG tank.

**Figure 16:**
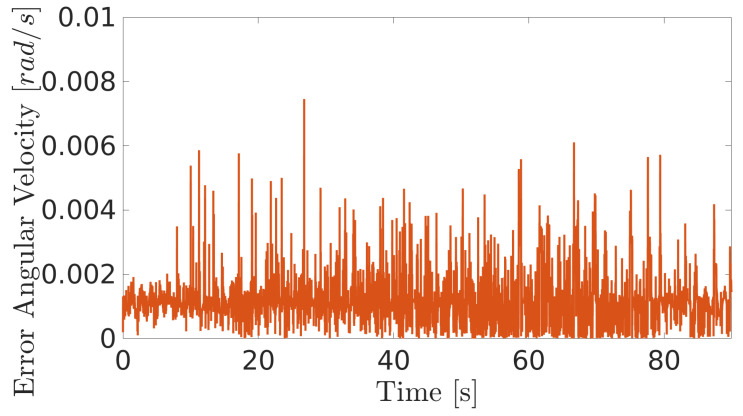
Angular velocity error of the robot when climbing the inner surface of the LPG tank.

The next experiment aims to highlight the robustness of the proposed controller during the climb, as well as in [17,38,39]. The climbing robot will navigate from the bottom to the top of the LPG tank’s inner surface at a constant speed (v=0.2 m/s and ω= 0 rad/s), as shown in Figure 14. As in the previous results, the controller reaches a good response, while generating negligible errors in speeds (Figure 15 and Figure 16). The proposed MPC controller commands the torque of the wheels to minimize the error of the robot’s speed about the reference speeds for its navigation. A supplementary video clip reproduces the robot’s climb on the inner surface of the tank.

**Figure 17:**
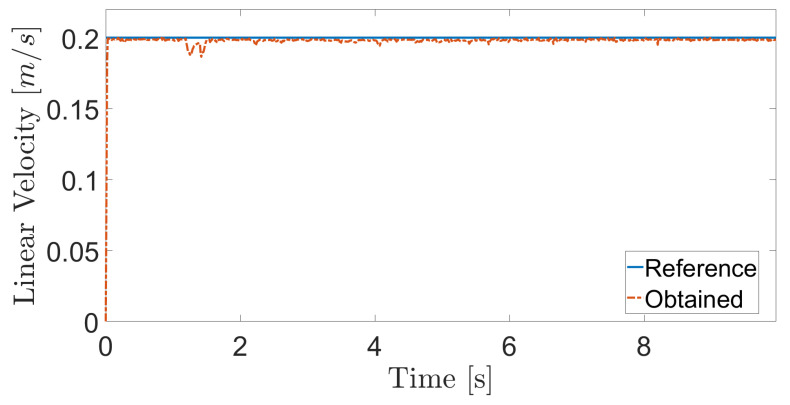
Linear velocity in the adhesion loss experiment.

**Figure 18:**
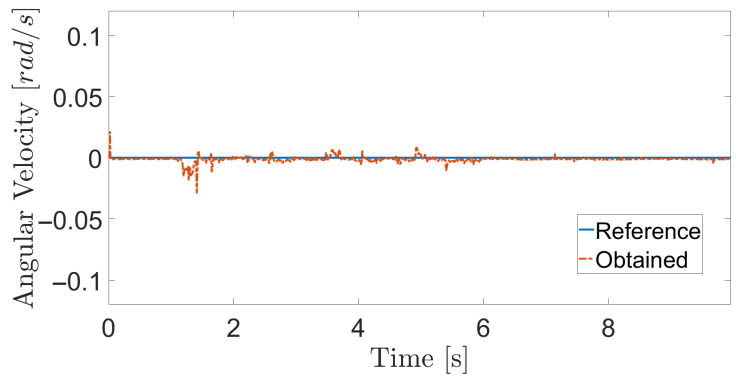
Angular velocity in the adhesion loss experiment.

Finally, a loss of adhesion test is carried out to assess the performance of the proposed controller. The loss of adhesion occurs during a short time (around 5 s) and represents 30% of the robot’s total adhesion. It can be caused by dirt on the metallic surface. The obtained performance of the MPC controller can be seen in Figure 17 and Figure 18. As expected, the robot loses velocity since the torque has decreased, beginning at 1s and during until 6 s. However, aside a small variation in speed around 2 s, there is no significant variation in velocity. The robot does not stop the climbing, since torque is maintained in the motors. During inspection, the robot’s adhesion to the metal surface is critical, since adhesion loss can cause the robot fall, hurting its operator or damaging the inspection equipment. Thus, some variation in speed can be accepted due to the need for the adhesion system to prevent the robot from falling when it passes over dirt, a weld bead or any surface flaw.

### 7.3. Controller Performance Analysis

The purpose of this section is to study the effect of varying surface elevation angle (0 to 180 degrees) on the MPC controller performance. Therefore, to validate the MPC controller in all situations that the robot may have, various experiments were carried out by varying the robot’s climbing angle on a flat metal plate with v=0.1 m/s and ω=0 rad/s for 5 s. The robustness analysis of the MPC controller is carried out considering the obtained results in seven different elevations given in Table 4.

This table gives us the average error and its standard deviation for both velocities at seven different elevations. In all situations, the MPC controller obtains an excellent performance in which medium error and standard deviation are insignificant. These good results confirm that the controller compensates the effects of gravity, adhesion, friction force, and other dynamic effects (i.e., inertial forces) that are correctly taken into account in the dynamic and kinematic modeling.

**Figure 19:**
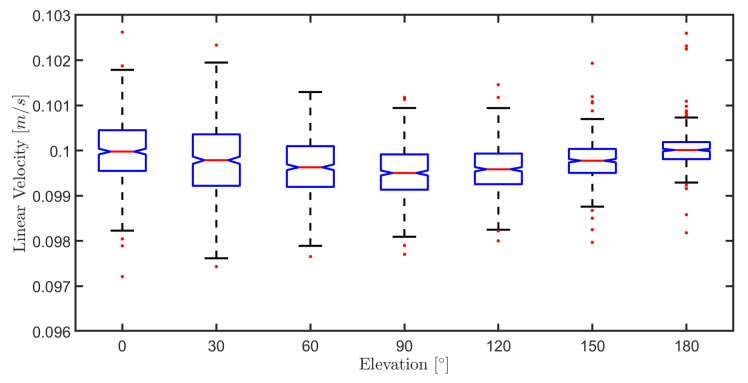
Boxplot of the linear velocities in the elevation variation experiment.

A behavioral analysis of linear velocities is gathered in the box plot from Figure 19. These plots show that there is a considerable variation in the robot’s linear speed at lower elevations. This is consistent with reality since in such cases, the torque needs to compensate for the interaction force (adhesion and friction) of the robot with the surface and gravity. This variation is more significant at angles between 0 and 60 degrees. However, as the elevation increases, the reaction force decreases, decreasing the controller’s effort. Besides, static and dynamic friction are not considered in the modeling.

**Figure 20:**
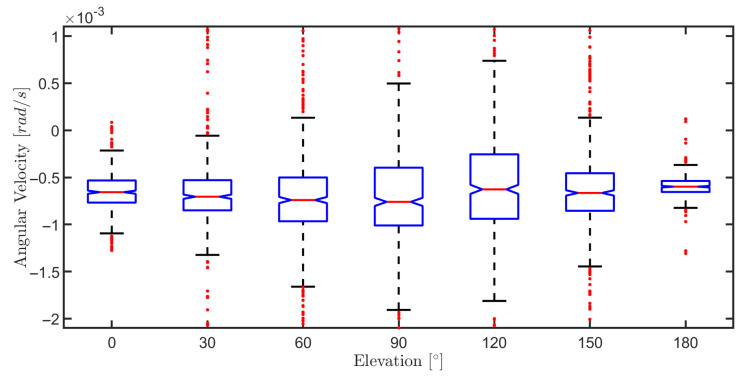
Boxplot of the angular velocities in the elevation variation experiment.

On the other hand, there is a minor variation in the robot’s angular speed, as illustrated in Figure 20. However, this variation increases for larger angles, indicating the effects of gravity and the interaction force on the controller. On the ground, gravity acts perpendicular to the robot’s plane, basically influencing the robot’s linear speed. But during the climb, the robot’s reaction force with the surface is weakened due to gravity’s influence, requiring a higher effort from the controller to maintain the same angular speed.

## 8. Experimental Results

The MPC controller is embedded in the real climbing robot shown in Figure 1 and Figure 2. This controller will be evaluated during experiments in which this robot develops climbing paths over a rotational super duplex plate, specially designed for these analyses. The super duplex stainless steel is a metal composite usually used in industrial vessels due to its high resistance to corrosion. However, such material has low magnetic adherence compared with standard steel, around 50% reduction, as discussed in [40]. The super duplex also presents a very high mechanical strength that results in a heavy platform harder to maneuver. Thus, a robust mechanical device was designed to rotate the super duplex plate, whose size is around 2 × 3 m, and weigh around 350 kg, to standard angles. This mechanism has three locks (0, 45, and 90 degrees) to preserve the turning link’s mechanical resistance, assigning a rigorous spacing between the holes. The speed reference of the MPC controller is v=0.02 m/s and ω=0 rad/s, and the surface is tilted 0, 45, and 90 degrees, as shown in Figure 1.

**Figure 21:**
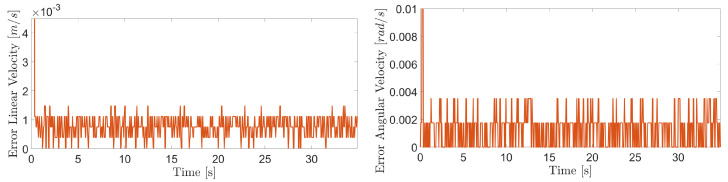
Linear and angular velocity errors during navigation of the climbing robot at 0 degree inclination.

**Figure 22:**
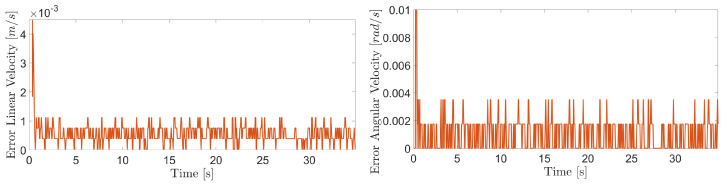
Linear and angular velocity errors during navigation of the climbing robot at 45 degree inclination.

**Figure 23:**
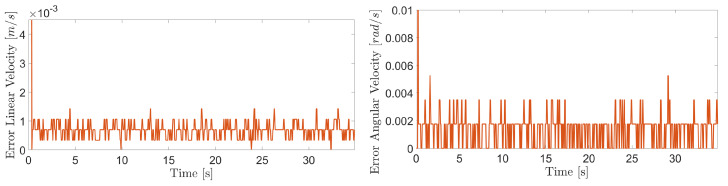
Linear and angular velocity errors during navigation of the climbing robot at 90 degree inclination.

The proposed controller presents a reliable performance in the experimental results with similar behavior to evaluations in the virtual environment. The Figure 21, Figure 22 and Figure 23 show the controller’s effectiveness. The velocity tracking error is calculated from the absolute value of the difference between the reference and the robot speed obtained from measurements through the forward kinematics. This error is irrelevant (around 8.46 × 10${}^{-4}$ m/s and 1.3 × 10${}^{-3}$ rad/s at 90${}^{\circ }$ of inclination) and it does not affect robot’s navigation. Mainly, it is not perceived by inspection equipment. In this way, the proposed controller testifies its ability to compensate for the effects of gravity, friction, and adhesion during the climb.

## 9. Conclusions

This work has discussed the kinematic and dynamic analysis of differential climbing robots. The navigation of a climbing robot presents disturbances in its dynamic parameters, due to gravity, adhesion and friction forces, which cannot be disregarded in the design of motion controllers. The Lagrange method was used to develop a complete model to a climbing robot encompassing all dynamic effects due to cited forces. The main goal was to get a more reliable model allowing a better control design to predict and compensate the disturbances caused by adhesion with the surface.

In the developed dynamic model, the effects of the forces of gravity, adhesion and friction were analyzed. The gravity force was modeled according to the robot’s spatial orientation because it can vary during its navigation. Meanwhile, the interaction force concept was introduced that represents the interaction between the contact surface and the robot’s wheels about friction, adhesion, and gravity.

A state-space model with integrator action was derived from the developed dynamic model that allowed the design of a Model Predictive Controller (MPC) to velocity tracking problem. Therefore, the controller can minimize the effects of unmodulated dynamic disturbances. This compensation for unmodeled disturbances is significant, since one of the applications of the climbing robot is to inspect storage tanks. During an inspection, the robot must have an accurate speed control with the smallest possible error ensuring that it navigates along the planned trajectory and correctly inspect the entire surface of the tank.

The presented results, as expected, have showed that a climbing robot does not need a very high torque to move on the ground. However, even higher torque is required during the climbing movement because the robot must compensate for the effects of inertia, friction, adhesion, and gravity.

The simulated results have highlighted the effectiveness of the proposed approach. For all essayed inclinations, the developed MPC controller has compensated for the dynamic effects existing during robot’s climb path. The practical results reaffirm the performance of the MPC controller in a real robot. The experiments show that dynamic modeling considering gravity, friction, and adhesion in the climbing robot was crucial to compensate for these dynamic effects.

The proposed MPC controller has performed well. However, we intend, as future work, to develop a model that considers static and dynamic friction, which allows us to minimize other error in the robot’s speed. This error may be related to the uncertainties in the identified model parameters, the material surface characteristics, and the non-measurable disturbances present in the real system.

## Figures and Tables

**Table 1:** Forces in climb interaction.

Force	Description
Normal ($F_{N}$)	reaction to robot compression over surface, that is always perpendicular to the robot’s contact plane and affects its adhesion.
Adhesion ($F_{A}$)	must ensure that the climbing robot can navigate over the entire surface without losing surface contact (i.e., must cancel gravitational effects). Adhesion force and normal force are always opposite as seen in situations exemplified in Figure 1.
Friction ($F_{F}$)	imposes a resistance to robot motion due to wheels friction, emphasized by gravitational force in some climbing situations.
Gravity ($F_{G}$)	affects the whole interaction between climbing robot and surface in several ways, according to robot position and motion over the surface. Gravity can increases or decreases the friction and adhesion forces.

**Table 2:** Robots parameters for numerical simulation.

$m_{P}=6$ kg	$m_{w}=1$ kg	b=0.5 m
r=0.08 m	l=0.5 m	c=0.5 m
a=0.5 m	$F_{ad}=40$ N	d=0.5 m
$I_{P}=0.1042$ kg·m/s${}^{2}$	$I_{m}=0.0016$ kg·m/s${}^{2}$	e=0.01 m
$I_{w}=0.0032$ kg·m/s${}^{2}$	$t_{w}=0.02$ m	f=0 m

**Table 3:** Tuning parameters of MPC.

Parameter	Specification
Sample time	0.01 s
Control Horizon	2
Prediction Horizon	5
Manipulated variable limits	[−10,10]
Velocity output limits	[−5,5]
Error output limits	[−10,10]
Weights on output	20
Weights on manipulated variables	0.2
Weights on manipulated variables rates	0.1

**Table 4:** Controller performance for robot navigation at different elevations.

Elevation	Linear Velocity [m/s]		Angular Velocity [rad/s]	
	Mean Error	Error Standard Deviation	Mean Error	Error Standard Deviation
$0^{\circ }$	$1.9\times 10^{-4}$	$4.27\times 10^{-3}$	$6.58\times 10^{-4}$	$2.42\times 10^{-4}$
$30^{\circ }$	$4.55\times 10^{-4}$	$4.58\times 10^{-3}$	$6.94\times 10^{-4}$	$5.99\times 10^{-4}$
$60^{\circ }$	$6.8\times 10^{-4}$	$4.87\times 10^{-3}$	$6.7\times 10^{-4}$	$1.79\times 10^{-4}$
$90^{\circ }$	$9.16\times 10^{-4}$	$4.71\times 10^{-3}$	$4.64\times 10^{-5}$	$5.99\times 10^{-3}$
$120^{\circ }$	$7.64\times 10^{-4}$	$4.85\times 10^{-3}$	$3.19\times 10^{-4}$	$6.86\times 10^{-3}$
$150^{\circ }$	$4.73\times 10^{-4}$	$4.6\times 10^{-3}$	$5.05\times 10^{-4}$	$1.94\times 10^{-3}$
$180^{\circ }$	$2.18\times 10^{-4}$	$4.55\times 10^{-3}$	$6.47\times 10^{-4}$	$1.16\times 10^{-3}$

## Appendix A

The robot’s motion matrices using the Lagrange-Euler approach.

(A1) $$\begin{array}{c} M\left(q\right)=\left[\begin{array}{ccccccc} m & 0 & M_{1} & 0 & 0 & 0 & 0\\ 0 & m & M_{2} & 0 & 0 & 0 & 0\\ M_{1} & M_{2} & I & 0 & 0 & 0 & 0\\ 0 & 0 & 0 & I_{w} & 0 & 0 & 0\\ 0 & 0 & 0 & 0 & I_{w} & 0 & 0\\ 0 & 0 & 0 & 0 & 0 & I_{w} & 0\\ 0 & 0 & 0 & 0 & 0 & 0 & I_{w} \end{array}\right] \end{array}$$

$$M_{1}=-m_{P}\left(e\sin(\theta )+f\cos(\theta )\right)-m_{w}\left(a-b-c+d\right)\sin\left(\theta \right)$$

$$M_{2}=m_{P}\left(e\cos(\theta )-f\sin(\theta )\right)+m_{w}\left(a-b-c+d\right)\cos\left(\theta \right)$$

(A2) $$\begin{array}{c} V\left(q,\dot{q}\right)=\left[\begin{array}{ccccccc} 0 & 0 & -m_{P}\dot{\theta }\left(e\cos(\theta )-f\sin(\theta )\right)-m_{w}\dot{\theta }\left(a-b-c+d\right)\cos\left(\theta \right) & 0 & 0 & 0 & 0\\ 0 & 0 & -m_{P}\dot{\theta }\left(e\sin(\theta )+f\cos(\theta )\right)-m_{w}\dot{\theta }\left(a-b-c+d\right)\sin\left(\theta \right) & 0 & 0 & 0 & 0\\ 0 & 0 & 0 & 0 & 0 & 0 & 0\\ 0 & 0 & 0 & 0 & 0 & 0 & 0\\ 0 & 0 & 0 & 0 & 0 & 0 & 0\\ 0 & 0 & 0 & 0 & 0 & 0 & 0\\ 0 & 0 & 0 & 0 & 0 & 0 & 0 \end{array}\right] \end{array}$$

(A3) $$\begin{array}{c} B\left(q\right)=\left[\begin{array}{cccc} 0 & 0 & 0 & 0\\ 0 & 0 & 0 & 0\\ 0 & 0 & 0 & 0\\ 1 & 0 & 0 & 0\\ 0 & 1 & 0 & 0\\ 0 & 0 & 1 & 0\\ 0 & 0 & 0 & 1 \end{array}\right] \end{array}$$

(A4) $$\begin{array}{c} A^{T}\left(q\right)\lambda =\left[\begin{array}{ccccccc} -\sin(\theta ) & \cos(\theta ) & 0 & 0 & 0 & 0 & 0\\ \cos(\theta ) & \sin(\theta ) & a-e & -r & 0 & 0 & 0\\ \cos(\theta ) & \sin(\theta ) & -b+e & 0 & -r & 0 & 0\\ \cos(\theta ) & \sin(\theta ) & -c+e & 0 & 0 & -r & 0\\ \cos(\theta ) & \sin(\theta ) & d-e & 0 & 0 & 0 & -r \end{array}\right]\left[\begin{array}{c} \lambda_{1}\\ \lambda_{2}\\ \lambda_{3}\\ \lambda_{4}\\ \lambda_{5}\\ \lambda_{6}\\ \lambda_{7} \end{array}\right] \end{array}$$

(A5) $$\begin{array}{c} \tau =\left[\begin{array}{c} \tau_{1}\\ \tau_{2}\\ \tau_{3}\\ \tau_{4} \end{array}\right] \end{array}$$

## Appendix B

The state space matrices for the proposed MPC controller.

(A6) $$\begin{array}{c} A_{ss}=\left[\begin{array}{cccccccc} -\bar{M}{\left(q\right)}^{-1}\bar{V}\left(q,\dot{q}\right) & 0 & 0 & 0 & 0 & 0 & 0 & 0\\ 0 & -\bar{M}{\left(q\right)}^{-1}\bar{V}\left(q,\dot{q}\right) & 0 & 0 & 0 & 0 & 0 & 0\\ 0 & 0 & -\bar{M}{\left(q\right)}^{-1}\bar{V}\left(q,\dot{q}\right) & 0 & 0 & 0 & 0 & 0\\ 0 & 0 & 0 & -\bar{M}{\left(q\right)}^{-1}\bar{V}\left(q,\dot{q}\right) & 0 & 0 & 0 & 0\\ -1 & 0 & 0 & 0 & 0 & 0 & 0 & 0\\ 0 & -1 & 0 & 0 & 0 & 0 & 0 & 0\\ 0 & 0 & -1 & 0 & 0 & 0 & 0 & 0\\ 0 & 0 & 0 & -1 & 0 & 0 & 0 & 0 \end{array}\right] \end{array}$$

(A7) $$B_{ss}=\left[\begin{array}{cccc} 1 & 0 & 0 & 0\\ 0 & 1 & 0 & 0\\ 0 & 0 & 1 & 0\\ 0 & 0 & 0 & 1\\ 0 & 0 & 0 & 0\\ 0 & 0 & 0 & 0\\ 0 & 0 & 0 & 0\\ 0 & 0 & 0 & 0 \end{array}\right]$$

(A8) $$C_{ss}=\left[\begin{array}{cccccccc} 1 & 0 & 0 & 0 & 0 & 0 & 0 & 0\\ 0 & 1 & 0 & 0 & 0 & 0 & 0 & 0\\ 0 & 0 & 1 & 0 & 0 & 0 & 0 & 0\\ 0 & 0 & 0 & 1 & 0 & 0 & 0 & 0\\ 0 & 0 & 0 & 0 & 1 & 0 & 0 & 0\\ 0 & 0 & 0 & 0 & 0 & 1 & 0 & 0\\ 0 & 0 & 0 & 0 & 0 & 0 & 1 & 0\\ 0 & 0 & 0 & 0 & 0 & 0 & 0 & 1 \end{array}\right]$$
